# Ubiquitination in Plant Meiosis: Recent Advances and High Throughput Methods

**DOI:** 10.3389/fpls.2021.667314

**Published:** 2021-04-07

**Authors:** Jamie N. Orr, Robbie Waugh, Isabelle Colas

**Affiliations:** ^1^Cell and Molecular Sciences, The James Hutton Institute, Dundee, United Kingdom; ^2^School of Life Sciences, University of Dundee, Dundee, United Kingdom; ^3^School of Agriculture and Wine, University of Adelaide, Adelaide, SA, Australia

**Keywords:** meiosis, ubiquitin, plant, HEI10, APC/C, SCF

## Abstract

Meiosis is a specialized cell division which is essential to sexual reproduction. The success of this highly ordered process involves the timely activation, interaction, movement, and removal of many proteins. Ubiquitination is an extraordinarily diverse post-translational modification with a regulatory role in almost all cellular processes. During meiosis, ubiquitin localizes to chromatin and the expression of genes related to ubiquitination appears to be enhanced. This may be due to extensive protein turnover mediated by proteasomal degradation. However, degradation is not the only substrate fate conferred by ubiquitination which may also mediate, for example, the activation of key transcription factors. In plant meiosis, the specific roles of several components of the ubiquitination cascade—particularly SCF complex proteins, the APC/C, and HEI10—have been partially characterized indicating diverse roles in chromosome segregation, recombination, and synapsis. Nonetheless, these components remain comparatively poorly understood to their counterparts in other processes and in other eukaryotes. In this review, we present an overview of our understanding of the role of ubiquitination in plant meiosis, highlighting recent advances, remaining challenges, and high throughput methods which may be used to overcome them.

## Introduction

### Meiosis

Meiosis is the production of haploid gametes through one round of DNA replication followed by two successive rounds of cell division. Meiotic recombination is the foundation of plant breeding efforts—essential to global food security—which seek to increase yield, drought tolerance, or resistance to pathogens in response to pressures on the food system such as global warming and a growing population. During the first meiotic division, replicated parental chromosomes—consisting of sister chromatids bound together by a ring-like complex called cohesin—condense, form homologous pairs, and are linked by a specialized tripartite protein structure called the synaptonemal complex (SC). Pairing is facilitated by the formation of double strand breaks (DSBs) in looped chromatin fibers, universally catalyzed by the conserved topoisomerase Spo11 (Bergerat et al., 1997; Keeney et al., 1997; Grelon et al., 2001), in conjunction with several other protein subgroups (Cole et al., 2010). DSB formation begins the process of meiotic recombination which is a result of their repair following partial 5′–3′ degradation (resection) of one strand of DNA at both sides of the break, yielding 3′-ended single stranded DNA (Osman et al., 2011; Mercier et al., 2015; Wang and Copenhaver, 2018; Pyatnitskaya et al., 2019). DSBs may be resolved as class I or class II crossovers (COs) or as non-crossovers (NCOs); NCOs being much more common than COs (Franklin et al., 1999; Copenhaver et al., 2002; Mercier et al., 2005). Considerable progress has been made in dissecting the timing, movement, and proteins which are involved in meiotic division, and their effects on recombination. The critical function of post-translational modifications (PTMs) in the regulation of meiotic division and recombination in eukaryotes is well-established (Sawada et al., 2014). One of the most abundant PTMs of proteins is ubiquitination, the covalent attachment of the 76 amino acid protein ubiquitin to target proteins (Ciehanover et al., 1978; Swatek and Komander, 2016). Ubiquitination regulates almost all cellular processes (Dye and Schulman, 2007). During meiosis, chromosome axes show extensive ubiquitination (Rao et al., 2017; Li Y. et al., 2018), while specific ubiquitin cascade interactions are required for key processes such as homologous recombination (Ward et al., 2007; Chelysheva et al., 2012; Wang et al., 2012) and chromosome segregation (Wang et al., 2013; Jonak et al., 2017; Kernan et al., 2018; Yamano, 2019).

### Ubiquitination

**FIGURE 1:**
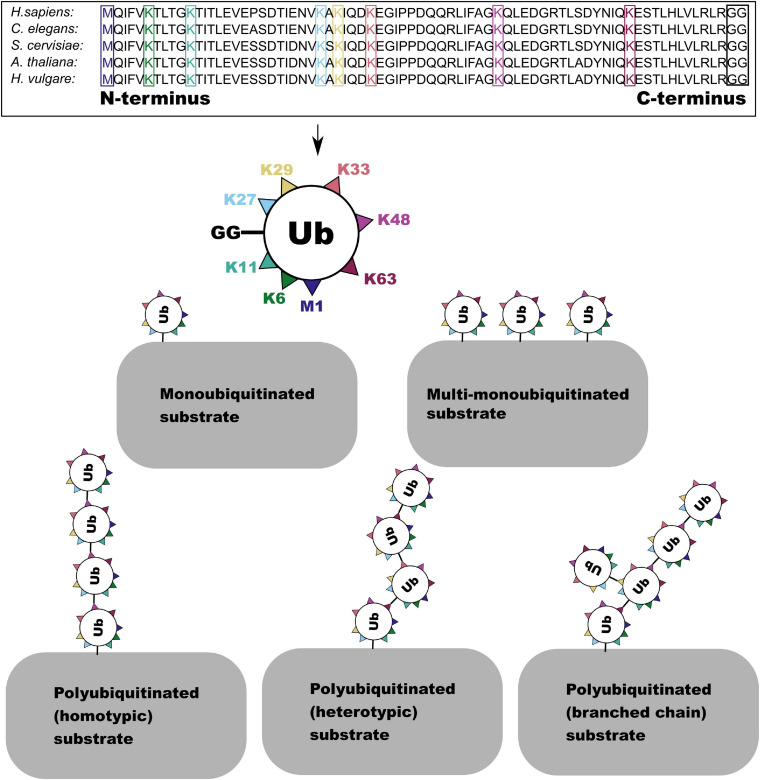
The amino acid sequence of the ubiquitin monomer is highly conserved across eukaryotes. Here the one N terminal methionine (M) and seven lysine (K) residues in the sequence which are able to form linkages with the C-terminal GG residue (boxed in black) are highlighted. This enables the formation of several forms of ubiquitin and polyubiquitin conjugate.

Ubiquitin shows remarkable conservation in the evolutionary history of eukaryotes, while the ubiquitination cascade has undergone massive expansion, resulting in one of the most versatile protein PTMs (Dye and Schulman, 2007; Zuin et al., 2014). This versatility derives from the ability of ubiquitin to form linked chains (polyubiquitination) *via* attachment of its C-terminal di-glycine motif (GG) to another ubiquitin protein at one of seven lysine (K6, K11, K27, K29, K33, K48, and K63) residues or to an N terminal methionine residue (M1) (Kulathu and Komander, 2012; López-Mosqueda and Dikic, 2014). In addition to polyubiquitination, proteins can be mono- or multi-monoubiquitinylated with unlinked ubiquitin (Emmerich and Cohen, 2015). Ubiquitin chains can be extended by a single linkage type or by multiple linkage types which may be formed at multiple residues on the same ubiquitin molecule forming a branched chain (Figure 1; Swatek and Komander, 2016). Ubiquitin can also be directly modified—in addition to the attachment of further ubiquitin to generate chains—by acetylation, phosphorylation, and attachment of ubiquitin-like modifiers (Swatek and Komander, 2016).

The canonical function of protein ubiquitination is to target the substrate for degradation by the proteasome, first described by Ciehanover et al. (1978). However, ubiquitin chain topology can confer specific substrate fates other than proteasomal degradation including recruitment of binding partners (Huang and D’Andrea, 2010), activation (Xu et al., 2009), or nuclear uptake (Plafker et al., 2004). Ubiquitination of a target protein is a tightly controlled cascade of ubiquitin activation, conjugation, and ligation involving three enzymes of increasing abundance and specificity—E1 activating enzymes, E2 conjugating enzymes, and E3 ligases (Dye and Schulman, 2007). E1 ubiquitin activating enzymes hydrolyze ATP forming an AMP-ubiquitin intermediate (Hatfield et al., 1997). The E1 enzyme then displaces AMP to form a thioester linkage to ubiquitin between an internal cysteine residue in the E1 and the carboxyterminal glycine of ubiquitin (Hatfield et al., 1997). The ubiquitin thioester bond is then transferred from the E1 activating enzyme to a cysteine residue in the ubiquitin conjugating (UBC) domain of an E2 conjugating enzyme (Ramadan et al., 2015). E3 ligases recruit ubiquitin conjugated E2s and target substrate proteins, conferring substrate specificity to the ubiquitination cascade (Iconomou and Saunders, 2016). E3 ligases can be divided into really interesting new gene (RING)/U-box, RING-in-between-RING (RBR), and homologous to E6AP C-terminus (HECT) domain containing groups (Dove et al., 2016). RING domain E3 ligases are the most abundant, binding both the substrate and E2-ubiquitin to catalyze the transfer of ubiquitin from E2 to the substrate protein (Dove et al., 2016). HECT E3s accept the transfer of the E2-thioester linkage forming an E3-ubiquitin intermediate before transferring ubiquitin to the substrate protein (Metzger et al., 2012). RBR E3 ligases are the least common and are characterized by the ordered appearance of a RING1 domain with a canonical structure, an in-between RING (IBR) domain, and a RING2 domain with a non-canonical RING structure (Dove et al., 2016). Although RBR E3s contain an E2-binding RING domain, they form a HECT-like E3-ubiquitin intermediate before transfer of ubiquitin to the substrate protein (Dove et al., 2016). The RING E3 ubiquitin ligases can be further subdivided into single and multi-subunit proteins (Iconomou and Saunders, 2016). An additional class of enzymes—E4 ubiquitin ligases—can extend shorter ubiquitin chains generated by E3 ligases (Hoppe, 2005). This can alter the fate of ubiquitinated protein from activation or transport to proteasomal degradation (Hoppe, 2005). Ubiquitination of substrate proteins by E3 and E4 ligases can also be trimmed or removed by deubiquitinating enzymes (DUBs), cysteine or metalloproteases which hydrolyze the bond between the modified protein and the C-terminal glycine of ubiquitin (Komander et al., 2009). Trimming or removal of ubiquitin can similarly alter substrate fate. The balance of E3/E4 and DUB activity can allow for fine tuning of protein activity as has been recently demonstrated in the acquisition of systemic acquired resistance in *Arabidopsis* (Huang et al., 2014; Skelly et al., 2019).

Ubiquitination seems to play an enhanced role in meiotic processes in all plants and higher eukaryotes. Transcriptome dynamics and characterization of a limited number of ligases indicates significant and varied roles for the ubiquitination cascade in plant meiosis which we are only beginning to explain. Although the identification of E3 substrate specificity is notoriously difficult, a number of tools are now available which may enable higher resolution characterization of such proteins, their target substrates, the types of ubiquitin chain linkages they build, and the roll of specific ubiquitination chain conformations in meiotic processes (Emmerich and Cohen, 2015; Iconomou and Saunders, 2016). Here we discuss recent developments in our understanding of ubiquitin—and ubiquitin like modifiers—in plant meiosis, with an emphasis on what is currently known about the role of specific E3 ubiquitin ligases and their substrates. Recent advances in mass spectrometry based molecular methods of identifying these interactions are also discussed in the context of their application to plant meiotic tissues.

## Transcriptome Dynamics Consistently Indicate an Enhanced Role for Ubiquitination in Plant Meiosis

**FIGURE 2:**
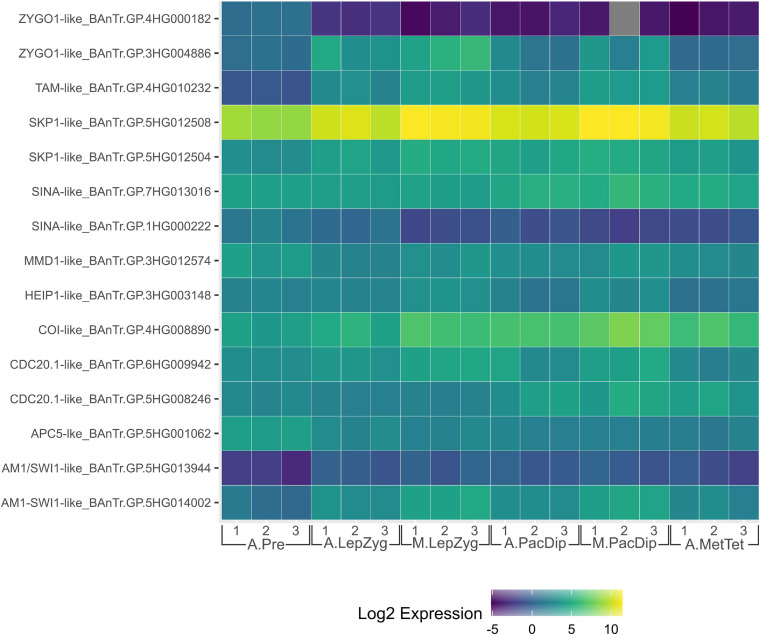
Heat map of expression of genes orthologous to those discussed in this review in the Barley Anther and Meiocyte Transcriptome (BAnTr) dataset. Three replicates each of anthers at pre-meiosis (A.Pre); anthers at leptotene–zygotene stages (A.LepZyg); meiocytes at leptotene–zygotene stages (M.LepZyg); anthers at pachytene–diplotene stages (A.PacDip); meiocytes at pachytene–diplotene stages (M.PacDip); and anthers at metaphase–tetrad stages (A.MetTet).

Enrichment of ubiquitin-proteasome system components is a common theme in plant meiotic transcriptome dynamics. In *Arabidopsis*, Yang et al. (2011) found that five of 17 Pfam domains significantly enriched in male meiocytes were related to ubiquitination. This was also reflected in the significant enrichment of the ubiquitination GO term (Yang et al., 2011). In our recent analysis of the barley anther meiotic transcriptome (BAnTr) dynamics we report significantly enriched expression of 71 potential E3 ligase genes in meiocytes, and differential expression of 166 putative E3 ligase genes before, during, or after prophase I in anthers (Barakate et al., 2021). Two genes orthologous to a *Drosophila melanogaster* seven *in absentia* (SINA) E3 ligase recently implicated in regulation of both assembly and disassembly of the SC (CG9949; Hughes et al., 2019), showed significant differential expression in barley prophase I (Figure 2). A further thirteen genes orthologous to E3 ligases or interactors with known roles in meiosis (discussed below) were present in the list of BAnTr differentially expressed genes (Figure 2).

**FIGURE 3:**
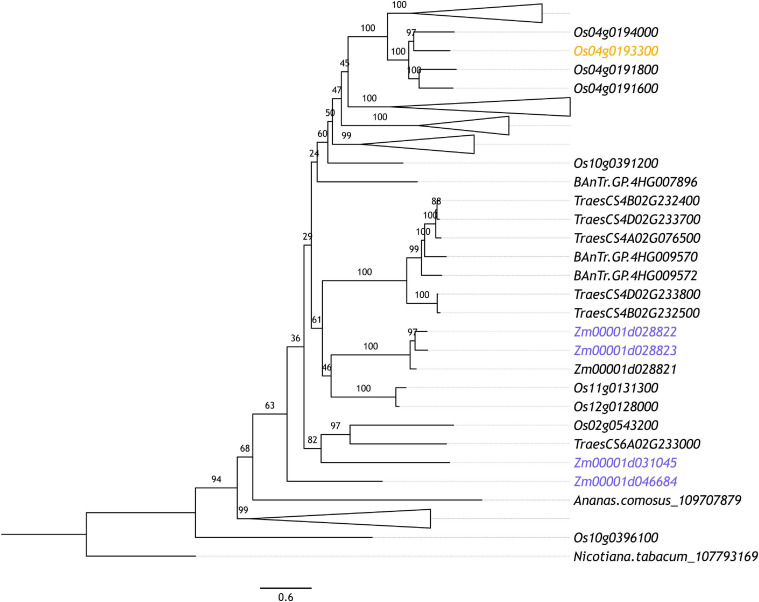
Maximum likelihood phylogenetic tree of orthologous maize and rice F-box genes whose expression is indicated to be up-regulated in PMCs according to Yuan et al. (2018) (highlighted in blue) and Tang et al. (2010) (highlighted in orange), respectively. Orthologous sequences were identified from tobacco (*Nicotiana tabacum*), rice (*Oryza sativa*), maize (*Zea mays*), pineapple (*Ananas comosus*), and Barley (*Hordeum vulgare*) Anther and Meiocyte Transcriptome (BAnTr) dataset using OrthoFinder (v.2.3.3; Emms and Kelly, 2015). The longest orthologous sequences from each species were aligned using MAFFT (v7.266; Katoh and Standley, 2013). Alignments were refined using Gblocks (v0.91b; Castresana, 2000). Maximum likelihood phylogeny was computed using IQ-TREE (v1.6.9; Nguyen et al., 2014) with ultrafast bootstrapping (*n* = 1,000). The resultant phylogeny was plotted using FigTree (v1.4.3). Branches are labeled with bootstrap support.

In maize, Yuan et al. (2018) reported that 39 genes preferentially expressed in pollen mother cells (PMCs) and 5 genes preferentially expressed in early PMCs (ePMCs) were E3 ubiquitin ligase components, including 18 F-box proteins in PMCs. F-box proteins confer substrate specificity as part of the multi-subunit SKP1-cullin_F-box (SCF) complex E3 ligases (Mocciaro and Rape, 2012), discussed in detail below. F-box proteins also appear to be enriched in rice meiotic tissues where Tang et al. (2010) identified 18 PMC enriched F-box-like genes. Interestingly, there is little crossover between these genes with only one of the PMC enriched F-box proteins in rice orthologous to those reported in maize. Further, this one rice F-box gene (Figure 3, highlighted in orange) is part of an expanded group of F-box-like genes in cereals which includes four of the 18 from maize but is far from the most similar rice ortholog to these four maize genes (Figure 3, highlighted in blue). This rice gene (Os04g0193300; F-box119) has no described role in replication or division but variants have been implicated in broad spectrum resistance to brown planthopper, an insect pest (Kamolsukyeunyong et al., 2019). This is the only characterization of any of the PMC preferentially expressed F-box genes in rice. Of the maize F-box genes, Zm00001d042833 (GRMZM2G125411; *ZmCOI1a*) is one of four maize orthologs of *CORONATINE INSENSITIVE (COI)-1* (An et al., 2018). The COI-1 protein is responsible for targeting the SCF complex to JAZMONATE ZIM-DOMIAIN 1, which binds to MYC transcription factors, repressing jasmonate responses (Thines et al., 2007; Yuan et al., 2018). In *Arabidopsis*, COI1 is required for male fertility (Xie et al., 1998). This is also true of its orthologs in maize which can rescue the infertility of *Arabidopsis* homozygous *coi1* mutants (An et al., 2018). Hence, COI1 enrichment in maize PMCs likely reflects increased jasmonate signaling pathway activity at the onset of meiosis. None of the 18 rice and maize F-box-like genes are orthologous to the F-box genes with characterized roles in plant meiosis, discussed below.

Taken together, these studies hint at the importance of ubiquitination to the regulation of plant meiosis. However, despite the vast number of ubiquitination related genes displaying differential expression in early meiosis, very few have been characterized. Currently, our understanding of the role of ubiquitination in this pathway is largely limited to a few extensively studied components: the SCF complex; the anaphase-promoting complex or cyclosome (APC/C); and human enhancer of invasion 10 (HEI10).

## SCF Complex E3s

**FIGURE 4:**
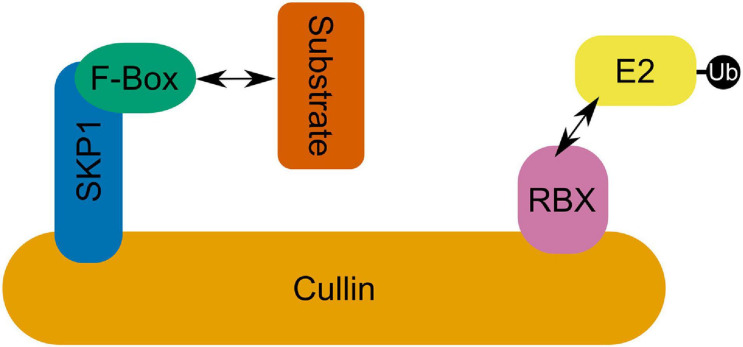
The SCF complex in which an E2 interacting RBX domain containing protein is linked via a Cullin to SKP1 which binds an F-box domain containing protein facilitating SCF complex-substrate interactions.

SCF RING E3 ubiquitin ligase complexes consist of a conserved modular format where an E2 binding Ring-box protein (RBX) is linked *via* a cullin (CUL1) scaffolding protein to an S-phase kinase-associated adaptor protein (SKP) which in turn binds a substrate recognition F-Box protein (Figure 4; Mocciaro and Rape, 2012). F-box proteins are the most varied group in this complex and are the most significant determinant of substrate specificity (Mocciaro and Rape, 2012). In fact, the F-box protein superfamily is one of the largest and most diverse in plants, although there is dramatic inter- and intra-specific variation in their number that is seemingly untethered to habitat or evolutionary history (Hua et al., 2011). *Arabidopsis* encodes 21 SKP1-like (ASK) proteins (Risseeuw et al., 2003). Among these, ASK1 and ASK2 are the most similar to SKP1 genes in yeast and humans—sharing 75% amino acid identity—and are able to interact with the same F-box proteins (Gagne et al., 2002; Kong et al., 2004). ASK1 is essential for *Arabidopsis* male fertility and synapsis (Yang et al., 1999; Wang and Yang, 2006). Transposon mutagenesis of ASK1 results in very stable association of homologous chromosomes which fail to separate at male anaphase I and remain associated at anaphase II despite normal spindle formation (Yang et al., 1999). ASK1 is also essential for the release of chromatin from the nucleolus which maintains a central location in mutants, failing to migrate to the nuclear periphery (Yang et al., 2006). Further, ASK1 appears to repress recombination as heterozygous ASK1/*ask1-1* plants demonstrate a recombination frequency approximately 2.6-fold greater than that of the wild type ASK1/ASK1 homolog (Wang and Yang, 2006). Despite the similarity of ASK1 and ASK2, *ask2* mutants are indistinguishable from wild type plants, showing no developmental defects (Liu et al., 2004). However, both ASK1 and ASK2 proteins are required for defective embryogenesis suggesting that they are in fact functionally redundant (Liu et al., 2004). The severity of the *ask1* single mutant in male meiosis seems to derive from the fact that while ASK1 is expressed in early prophase I anthers, ASK2 is not (Wang and Yang, 2006); while in developing embryos both ASK1 and ASK2 are expressed, allowing ASK2 to compensate for *ask1* mutants (Liu et al., 2004). Analysis of various ASK genes highlights diverse and overlapping expression patterns in organs and tissues as well as specific F-box interactions (Marrocco et al., 2003; Risseeuw et al., 2003; Takahashi et al., 2004; Dezfulian et al., 2012). Expression of wheat SKP1-like gene TSK1 in *Arabidopsis ask1*-*1*/*ask1*-*1* mutants was capable of partially rescuing of the sterile phenotype (Li et al., 2006). Recent evidence in mice—which along with humans and yeast possess only one *SKP1* gene—shows that SKP1 localizes specifically to the lateral element of the SC in spermatocytes where synapsis is complete (Guan et al., 2020). Further, germ cell specific inactivation of SKP1 in mouse testis led to the accumulation of HORMADs on the SC in pachytene and diplotene stages (Guan et al., 2020). Proteins of the HORMAD family regulate formation of DSBs and COs and their PCH2/TRIP13 mediated removal is involved in the coordination of SC assembly (Lambing et al., 2015; Vader, 2015). Recently, West et al. (2019) identified HORMAD-binding closure motifs in both mammalian and plant lateral element proteins SYCP2 and ASY3, indicating significant overlap in the mechanistic principle of meiotic chromosome axis assembly in eukaryotes. Guan et al. (2020) also showed that SKP1 depletion in mouse spermatocytes led to a concomitant decrease in TRIP13 abundance, speculating that SKP1 may be involved in stabilizing TRIP13. Given the conservation of SKP1-like protein sequence and apparent role in meiosis across eukaryotes (McLoud and Yang, 2012), it is tempting to speculate a common role for SCF complex mediated regulation of TRIP13/PCH2 in SC formation. However, as SKP1-like proteins may interact with multiple F-box proteins, phenotypic observations of SKP1-like protein meiotic mutants are likely to reflect multiple SCF E3 ligase complexes. Consequently, discovery and biochemical characterization of meiotic F-box proteins is a crucial step in continuing to unravel the role of SCF complexes in meiosis.

In rice, an F-Box protein called MEIOTIC F-BOX (MOF)—which interacts with rice SK1 ortholog OSK1—has been shown to be involved in the formation of the telomere bouquet, homologous chromosome pairing, synapsis, and DSB repair (He et al., 2016). MOF is highly expressed during meiosis and is active in leptotene to pachytene stage (He et al., 2016). *mof* mutants are completely male sterile, exhibiting arrested meiocyte development at late prophase I where chromosomes aggregate into a chromosome mass and degrade (He et al., 2016). Cytology of *mof* mutant meiocytes indicates severe disruption of SC formation and a lack of telomere clustering (He et al., 2016). Further, although phosphorylated H2AX foci appear normal at zygotene stage, indicating normal DSB formation, these foci are not reduced in number at pachytene stage, indicating that DSBs are not repaired (He et al., 2016). Immunolocalization showed that more than half of MOF foci colocalize with phosphorylated H2AX, and one third with COM1 and RAD51 indicating localization around DSB repair sites (He et al., 2016). A second rice F-Box protein, zygotene1 (ZYGO1), also interacts with OSK1 and has a putative role in meiosis (Zhang et al., 2017). Unlike *mof* and *ask1-1* mutants *zygo1* mutants are both male and female sterile (Zhang et al., 2017). ZYGO1 appears to regulate the formation of the telomere bouquet which does not form in the *zygo1* mutant (Zhang et al., 2017). *zygo1* mutants also demonstrate aberrant SC assembly with mutant SC length being 78.7% smaller than that of the wild type (Zhang et al., 2017). Further, although DSB and early recombination element installation is normal there is a significant reduction in cross-over (CO) formation (Zhang et al., 2017). In *Arabidopsis*, a plant specific F-box protein called COP9 signalosome interacting F_box Kelch 1 (CFK1), one of two highly similar CFK proteins in *Arabidopsis*, is also capable of forming an SCF complex (SCF^*CFK*1^; Franciosini et al., 2013). Recently, Chen et al. (2020) demonstrated that SCF^*CFK*1^ interacts directly with domains rearranged methyltransferase 2 (DRM2) which catalyzes CHH methylation of euchromatin—predominantly transposable elements (TEs)—guided by 24nt siRNAs through the small RNA-directed DNA methylation (RdDM) pathway (Matzke and Mosher, 2014). In meiosis, silencing of TEs *via* methylation is essential to ensuring genetic integrity in progeny (Hsieh et al., 2016; Walker et al., 2018). Overexpression of CFK also led to a small decrease in CHH type methylation and a subsequent significant increase in expression of four hypomethylated TEs and genic regions (Chen et al., 2020). Despite this, no change in the total amount of ubiquitin-DRM2 ligation was observed between WT and *cfk1* null mutant lines (Chen et al., 2020).

## The Anaphase-Promoting Complex

The APC/C, like the SCF complex, is a multi-subunit E3 ligase with core cullin (APC2) and RING domain containing (APC11) subunits (Eloy et al., 2015). However, the APC/C complex is much more complex, comprising at least 11 subunits (Eloy et al., 2015). Human APC/C interacts with ubiquitin conjugating E2 S (UBE2S), the only known E2 ubiquitin conjugating enzyme involved in specific K11-linked chain assembly (Wickliffe et al., 2011; Min et al., 2015). Homotypic K11 chains have been shown to prevent association with the mammalian proteasome (Grice et al., 2015). However, human APC/C interacts with both UBE2C and UBE2S forming heterotypic chains of branched K48 and K11 linkage types which leads to faster substrate proteasomal degradation than homotypic K11 or K48 chains alone (Meyer and Rape, 2014; Grice et al., 2015; Min et al., 2015). In *Saccharomyces cerevisiae*, the APC/C assembles K48 chains on its substrates in conjunction with ubiquitin conjugating E2 1 (Ubc1) and rapidly monoubiquitinates substrates in conjunction with Ubc4 (Rodrigo-Brenni and Morgan, 2007). Unfortunately, little is known about such atypical ubiquitin chain linkages in plants (Walsh and Sadanandom, 2014). *Arabidopsis* UBE2S ortholog UBC22 may be able to form K11 linked chains in conjunction with the APC/C but this remains to be experimentally validated (Wang et al., 2016). Substrate recognition by the APC/C is reliant on the presence of one or more of four conserved motifs: destruction box (D-box), KEN-box, GxEN-box, and A-box (Glotzer et al., 1991; Pfleger and Kirschner, 2000; Littlepage and Ruderman, 2002; Castro et al., 2003). In plants the function of only D-box and KEN-box motifs in APC/C mediated proteasomal degradation is validated (Eloy et al., 2015).

The APC/C is critical for both male and female meiosis in *Arabidopsis* (Zheng et al., 2011; Wang et al., 2013). Activation and substrate specificity of the APC/C is determined by the related co-factors Cell Division Cycle 20 (CDC20) and Cell Cycle Switch Protein 52 (CCS52). There are five CDC20-like genes in *Arabidopsis*, of which two (AtCDC20.1 and AtCDC20.2) are expressed and functionally redundant in mitosis (Kevei et al., 2011). CDC20.1—which interacts with APC/C subunits APC3, APC8, and APC10 (Kevei et al., 2011; Qiao et al., 2016)—is essential to proper chromosomal segregation (Niu et al., 2015). Similarly, AtAPC8 has been shown to be involved in chromosome alignment, chromosomal segregation, and microtubule organization (Xu et al., 2019). In recent years, considerable progress has been made in understanding the precise role and substrate specificity of APC/C in chromosomal segregation at anaphase I and II in *Arabidopsis*.

**FIGURE 5:**
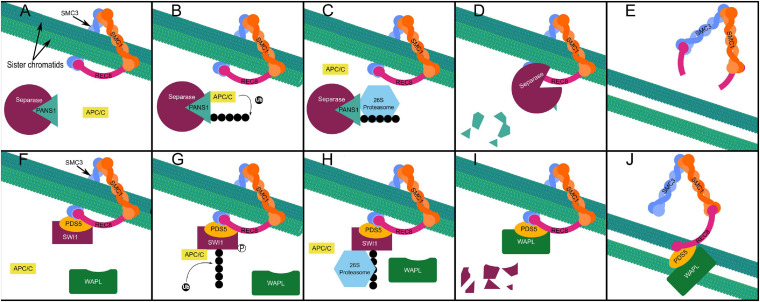
Proteolytic cohesin removal **(A–E)**. Separase is inhibited by PANS1 protecting REC8 from proteolytic cleavage at chromosome arms preceding anaphase I and at the centromere preceding anaphase II redundantly and in parallel with SGO (A), APC/C mediated ubiquitination of PANS1 **(B)**, triggers its proteasomal degradation **(C,D)**, freeing separase **(D)** to cleave Rec8, the kleisin subunit of the cohesin ring, allowing sister chromatid separation in anaphase II **(E)**. Non-proteolytic (prophase pathway) cohesin removal **(F–J)**. SWI1 associates with cohesin beginning at interphase and inhibits the interaction of WAPL with PDS5 **(F)**. Phosphorylation of SWI1 enables APC/C mediated ubiquitination **(G)**. Proteasomal degradation of ubiquitinated SWI1 **(H–I)**, enables WAPL to interact with PDS5 **(I)**, triggering non-proteolytic “opening” of the cohesin ring complex and sister chromatid release from the onset of zygotene stage to the end of pachytene stage in prophase I **(J)**.

Sister chromatid cohesion during the first meiotic division is maintained in part by Shugoshin (SGO), which recruits protein phosphatase 2A (PP2A) to dephosphorylate the meiotic kleisin subunit of cohesin—REC8—protecting it from cleavage by the evolutionarily conserved protease separase (Kitajima et al., 2004; Cromer et al., 2019). Degradation of SGO1 in yeast is triggered by ubiquitination by the APC/C at anaphase II, allowing sister chromatid segregation (Jonak et al., 2017). In *Arabidopsis*, PATRONUS1 (PANS1) acts independently and in parallel to SGO to prevent premature cleavage of centromeric cohesin at anaphase I (Cromer et al., 2019). PANS1 occupies the active site of separase until its proteasomal degradation frees separase to cleave REC8 (Figures 5A–E; Cromer et al., 2019). Abolishing the interaction of PANS1 with the APC/C also prevented homologous chromosome separation at anaphase I, indicating that some degradation of PANS1 is required prior to anaphase I to allow separase mediated removal of cohesin at chromosome arms (Cromer et al., 2019). A separate non-proteolytic pathway results in the removal of approximately 90% of cohesin is from chromosomes in late prophase I (Yang et al., 2019). Non-proteolytic cohesin removal by Wings Apart-Like (WAPL) occurs from the onset of zygotene stage to the end of pachytene stage (Figures 5F–J; Yang et al., 2019). Switch 1 (SWI1) binds to precocious dissociation of sister 5 (PDS5), a cohesin accessory protein which assists in the acetylation of the SMC3 subunit, preventing interaction of PDS5 with WAPL in early prophase I (Figure 5F; Yang et al., 2019). In zygotene stage, SWI1 is phosphorylated allowing its ubiquitination by the APC/C—interacting *via* five D-box domains—and subsequent proteasomal degradation (Figures 5G–I; Yang et al., 2019). This allows WAPL interaction with PDS5 resulting in dissociation of the kleisin subunit from SMC3, “opening” the cohesin ring and allowing it to dissociate from chromatin (Figure 5J; Yang et al., 2019). While non-proteolytic cohesin removal mediated by WAPL is essential for homologous chromosome segregation at anaphase I (Yang et al., 2019), in the absence of both SGO and PANS1 there is complete loss of cohesion at metaphase I, indicating that PANS1 and SGO also protect chromosome arm cohesin from separase (Cromer et al., 2019).

SWI1 possesses sequence similarity of approximately 30% with maize and rice ameiotic 1 (AM1), required for very many early meiotic processes including sister chromatid cohesion in maize (Pawlowski et al., 2009; Che et al., 2011). Interestingly, PANS1 is well conserved in dicots but not in monocots (Cromer et al., 2013). It has been hypothesized that the rice salt sensitivity1 (RSS1) gene represents a monocot PANS1 ortholog based on: positionally limited sequence similarity; shared N-terminal KEN and D-box domain architecture facilitating APC/C interaction; shared salt sensitivity of knockout mutants; and apparent meristematic cell cycle regulation by RSS1 (Ogawa et al., 2011; Cromer et al., 2013, 2019). However, defects in meiotic segregation have not been demonstrated in *rss1* mutants, which are both viable and fertile (Ogawa et al., 2011). Cromer et al. (2019) highlight the presence of an uncharacterized RSS1 paralog, possibly possessing redundant function, which could explain the lack of *rss1* infertility. Yeast two-hybrid assays appear to show interactions between PDS5A and AM1 (Yang et al., 2019) supporting the hypothesis that AM1 performs the same functional role to SWI1. However, as with the role of RSS1 or its paralog in chromosomal segregation, this remains to be experimentally validated.

Oscillation in cyclin dependant kinase (CDK) activity dictates the timing and directionality of the cell cycle in both meiosis and mitosis (Coudreuse and Nurse, 2010). CDKs and cyclins form complexes to drive DNA replication and cell division events through phosphorylation of substrates such as DMC1, REC8, and SPO11 (Wijnker and Schnittger, 2013). The amount and type of cyclin available to form cyclin-CDK complexes regulates their activity and substrate specificity (Pines, 1995; Harashima and Schnittger, 2012). The APC/C regulates CDK activity by targeting cyclins for degradation and is in turn regulated by several activator and inhibitory proteins (Bolanos-Villegas et al., 2018). Dysregulation of the APC/C through disruption of these proteins can result in premature termination of meiosis following the first division or failure to terminate leading to entry into a third cycle of division (Cromer et al., 2012). Consequently, the regulation by and of the APC/C at this stage is fundamental to meiosis.

Initiation of each meiotic division is reliant on CDK activity rising to cross a threshold—peaking at metaphase I and II—as APC/C activity is reduced (Wijnker and Schnittger, 2013). In *Arabidopsis*, loss of function of either of the cyclins ommission of second division (OSD1) or tardy asynchronus meiosis (TAM) results in premature exit from meiosis following the first division (D’Erfurth et al., 2010). Further, loss of function in both TAM and OSD1 leads to meiotic exit following prophase I without entry into the first division, producing tetraploid spores and gametes (D’Erfurth et al., 2010). OSD1 interacts directly with the APC/C activating subunits CDC20.1, CDC20.5, CCS52A1, CCS52A2, and CCS52B through its conserved D-BOX and MR-tail domains to inhibit APC/C activation (Iwata et al., 2011; Cromer et al., 2012). In between the first and second division CDK activity drops below the threshold which triggers the initiation of division as APC/C mediated proteasomal destruction of cyclins increases (Wijnker and Schnittger, 2013). APC/C activity must then drop to trigger spindle disassembly and to allow CDK activity to rise back above this threshold to initiate the second meiotic division (Wijnker and Schnittger, 2013). However, should APC/C activity rise too much between the first and second division this leads to the separation of sister chromatids and premature termination of meiosis as is observed in the OSD1 mutant (Azumi et al., 2002; D’Erfurth et al., 2010; Wijnker and Schnittger, 2013). Therefore, OSD1 functions to partially inhibit activation of the APC/C to allow CDK activity to fall to a level sufficient for spindle disassembly while preventing sister chromatid segregation (Cromer et al., 2012). OSD1 is not conserved in mammals or yeast although, as the APC/C activators are highly conserved, expression of OSD1 in mouse oocytes leads to arrested development at metaphase I (Cromer et al., 2012). TAM forms an active complex with CDKA;1, the major cell cycle CDK in *Arabidopsis* (Cromer et al., 2012; Nowack et al., 2012). CDKA;1 has been shown to regulate meiotic progression, sister chromatid cohesion, chromosome axis formation, the number and position of COs, and microtubule organization (Wijnker et al., 2019; Yang et al., 2019; Sofroni et al., 2020). CDKA;1-TAM complexes appear to control formation of the new cell wall between separated nuclei during division but not the meiotic spindle (Prusicki et al., 2019; Sofroni et al., 2020). Further, CDKA;1-TAM is proposed to inhibit the APC/C component three division mutant 1 (TDM1) at meiosis I (Cifuentes et al., 2016). *Arabidopsis* meiocytes carrying null mutant *tdm1* fail to exit meiosis, indicating that TDM1 modifies APC/C activity and/or specificity to trigger a reduction in CDK activity necessary for meiotic exit (Cifuentes et al., 2016). As TAM is expressed only in meiosis I and TDM1 is expressed throughout both meiosis I and II, premature exit from meiosis in *tam* mutants may be explained by the loss of CDKA;1-TAM inhibition of APC/C-TDM1 activity at metaphase I (Bulankova et al., 2010; Cifuentes et al., 2016).

## Hei10

HEI10 is an E3 ubiquitin ligase which is part of a family of structurally and functionally related proteins sharing an N-terminal RING domain (Chelysheva et al., 2012). Another notable member of this family is the ZMM protein ZIP3/RNF212 (Chelysheva et al., 2012). Plants and fungi encode only HEI10 (Chelysheva et al., 2012), whereas budding yeast, *Drosophila*, and *C. elegans* encode only ZIP3/RNF212 (Agarwal and Roeder, 2000; Jantsch et al., 2004), and vertebrates encode both (Qiao et al., 2014; de Muyt et al., 2014). In mice, HEI10 and RNF212 are not redundant but both cooperative and antagonistic (Qiao et al., 2014; Rao et al., 2017). The apparently divergent functions of HEI10 and ZIP3/RNF2121 in vertebrates is largely attributed their respective ubiquitin and small ubiquitin-like modifier (SUMO) ligase activity (Qiao et al., 2014; Rao et al., 2017). SUMOylation operates *via* a similar E1, E2, and E3 cascade as ubiquitination; but, unlike ubiquitin ligases, SUMO ligases are non-essential to substrate SUMOylation, and SUMO itself may bind non-covalently to proteins (Bernier-Villamor et al., 2002; Lin et al., 2006). In mice, both HEI10 and RNF212 have SUMO E3 ligase activity (Strong and Schimenti, 2010; Rao et al., 2017). However, RNF212 appears to act primarily as a SUMO ligase, which antagonizes the rate of HEI10 mediated substrate ubiquitination and destruction (Qiao et al., 2014; Rao et al., 2017). In contrast, HEI10 directly antagonizes RNF212 by promoting its proteasomal degradation (Qiao et al., 2014). However, both HEI10 and RNF212 are absolutely required for class I CO formation in mammals, which constitute 80–90% of total crossovers (Ward et al., 2007; Reynolds et al., 2013). In fact, the absolute requirement for HEI10 or ZIP3/RNF212 orthologs for class I CO formation is conserved in *Arabidopsis* (Chelysheva et al., 2012; Ziolkowski et al., 2017), *C. elegans* (Jantsch et al., 2004), *Sordaria* (de Muyt et al., 2014), and rice (Wang et al., 2012). In mouse spermatocytes, SUMO, ubiquitin, and the proteasome localize to the chromosome axes at zygotene stage (Rao et al., 2017). Chemical inhibition of ubiquitin activation, SUMO conjugation, and proteasomal degradation each led to a dramatic increase in SC central element proteins SYCP3 and SYP2 and defective synapsis (Rao et al., 2017). Further, ubiquitin and SUMO appeared interdependent, where inhibition of SUMO conjugation reduced association of both ubiquitin and the proteasome at chromosome axes; while SUMO accumulated on the axes when ubiquitin activation was inhibited; and both SUMO and ubiquitin accumulated when the proteasome was inhibited (Rao et al., 2017). However, while ubiquitin promotes proteasomal degradation of RAD51 and DMC1, SUMO appears to negatively regulate their rate of turnover (Rao et al., 2017). In contrast, inhibition of SUMO leads to the accumulation of HEI10 indicating negative regulation of HEI10 accumulation (Rao et al., 2017). As in mice, both ubiquitin and SUMO have been shown to localize to the chromosome axes in rice and *Arabidopsis* respectively (Li Y. et al., 2018; Lambing and Heckmann, 2018).

In addition to RNF212, several HEI10 substrate proteins in mammals have been identified. Mammalian HEI10, like the APC/C, regulates CDK dependant cell cycle progression by targeting B type cyclins for degradation (Singh et al., 2007; Ward et al., 2007). HEI10 also appears to mediate degradation of the RecA-related recombinase RAD51, but not DMC1, in mouse spermatocytes as well as ZMM proteins—which associate with and stabilize homologous recombination intermediates— MutSℽ (Msh4-Msh5), MER3, and TEX11 (Reynolds et al., 2013; Qiao et al., 2014; Rao et al., 2017). However, in a recent analysis of MutSℽ component Msh4 in yeast, which possesses only ZIP3, He et al. (2020) demonstrated that Msh4 was a target of the 20S proteosome, independent of ubiquitination, and could be stabilized by phosphorylation. Rao et al. (2017) hypothesize that, in mammals, the antagonistic activities of RNF212 and HEI10 determine the fate of recombination intermediates: where predominant RNF212 mediated SUMOylation of ZMM proteins in a minority of strand exchange intermediates results in class I crossover formation; while predominant HEI10 mediated ubiquitination of ZMMs results in formation of NCOs. In yeast and *C. elegans*, ZIP3 appears to act exclusively as a SUMO E3 ligase (Cheng et al., 2006; Bhalla et al., 2008). In the fungus *Sordaria macrospora*, HEI10 was shown to positively regulate SUMO localization to the SC *via* its RING domain (de Muyt et al., 2014).

In *Arabidopsis*, HEI10 appears as ∼100–200 foci in leptotene to early pachytene stage (Chelysheva et al., 2012). In late pachytene stage HEI10 foci dramatically reduce in number by ∼90% co-localizing with MLH1 (Chelysheva et al., 2012), which is involved in late recombination and class I crossover maturation (Hunter and Borts, 1997). Despite appearing as foci in early meiotic prophase I meiotic defects are not apparent until diakinesis in *hei10* mutants, corresponding to the disappearance of HEI10 foci in the wild type (Chelysheva et al., 2012). In addition to being required for their formation, in *Arabidopsis* HEI10 promotes class I COs in a dose dependant manner (Ziolkowski et al., 2017; Serra et al., 2018). Increasing the copy number of HEI10 in *Arabidopsis* was sufficient to more than double DSB resolution as COs (Ziolkowski et al., 2017). Further, increased HEI10 expression also increases crossover coincidence, indicating that HEI10 also plays a role in crossover interference (Serra et al., 2018). In rice, HEI10 was shown to be capable of forming multi-protein complexes with ZMM proteins ZIP4, PTD, SHOC1, and MSH5 (Zhang J. et al., 2019). Additionally, OsHEI10, OsZIP4, OsSHOC1, and OsPTD displayed variable interdependence in loading to the chromosome axis (Zhang J. et al., 2019). Li Y. et al. (2018) identified a plant specific protein called HEI10 interaction protein (HEIP1), which colocalizes with HEI10 on crossover sites from late pachytene to diplotene stage and is also required for class I CO formation. In addition to its interaction with HEI10, HEIP1 interacts directly with ZMM proteins ZIP4 and MSH5 (Li Y. et al., 2018). Further, loading of HEIP1 on chromosome axes was dependant on both HEI10 and ZIP4 (Li Y. et al., 2018). Chang et al. (2019) described a highly similar meiotic phenotype in their description of aberrant gametogenesis 1 (OsAGG1), which is synonymous with HEIP1. This work confirmed the essential role of OsAGG1/HEIP1 in class I CO formation as well as its interaction with HEI10, ZIP4, and MSH5 (Chang et al., 2019). However, Chang et al. (2019) also characterized four conserved N-terminal motifs which were essential to its function and interaction with characterized ZMMs.

## Ubiquitin-Like Modifiers

Related to ubiquitin (RUB) is another small peptide post-translational protein modifier in plants. In animals and fission yeast this modifier is known as neuronal precursor cell expressed developmentaly down-regulated 8 (NEDD8). The covalent attachment of this modifier to proteins is called rubylation or neddylation and is mediated by a cascade which— like sumoylation and ubiquitination—is dependent on specific RUB activating (E1), conjugating (E2), and ligating (E3) enzymes (Table 1). Jahns et al. (2014) demonstrated that *Arabidopsis* auxin resistant 1 (AXR1)—one half of the RUB E1 activating enzyme heterodimer (Leyser et al., 1993)—was involved in distribution of class I COs but not their number. Recently, Christophorou et al. (2020) expanded on this work to demonstrate a regulatory role for AXR1 in pericentromeric and transposable element methylation. Further, AXR1 deficient mutants exhibit enhanced sensitivity to DNA damage and significant down-regulation of HEI10, TOPII, and MLH3 (Martinez-Garcia et al., 2020). However, AXR1 acts upstream of E2 conjugating and E3 ligating enzymes, meaning that AXR1 mutant phenotypes might reflect defects in several distinct pathways. Indeed, the role of AXR1—and, by extension, rubylation—in regulating DNA methylation is not coupled to its role in determining CO distribution (Christophorou et al., 2020). Instead, CO abnormalities in *axr1* mutants are likely a product of aberrant synapsis due to a failure of ZYP1 to polymerize fully (Jahns et al., 2014). Disruption of CUL4 expression leads to a similar meiotic phenotype to *axr1* mutants indicating that the *axr1* meiotic phenotype might reflect perturbed CUl4 rubylation mediated by RBX1 which acts as both an E3 in the rubylation cascade and as part of SCF and cullin ring ligase 4 (CRL) ubiquitin E3 complexes (Jahns et al., 2014). In *C. elegans*, mutants of the CRL4 components CUL4 and DDB-1 also display aberrant synapsis with SYP-1, the ZYP1 equivalent, failing to polymerize normally, forming large polycomplexes (Brockway et al., 2014; Alleva et al., 2019). Interestingly, RBX1 mutants showed no defects in synapsis (Alleva et al., 2019). However, the role of CUL4 in SC formation does not appear to be universal as *cul4A* knockout mutants synapse fully in mouse spermatocytes (Kopanja et al., 2011; Yin et al., 2011). The meiotic substrates of CRL4 have yet to be identified, hindering the development of a molecular mechanistic model of its interaction in SC formation and DNA repair. Like SKP1, AXR1 appears to have undergone gene duplication in plants; however, unlike ASK2, AXR1-LIKE (AXL), sharing 80% amino acid identity with AXR1, was shown not to possess redundant function with AXR1 in meiosis, although it did also display a role in DNA damage repair (Martinez-Garcia et al., 2020).

**TABLE 1:** Comparison of the number of identified E1, E2, and E3 enzymes in the ubiquitination, rubylation, and sumoylation cascade in *Arabidopsis* and processes they are known to regulate in meiosis.

	**E1**	**E2**	**E3**	**Described regulation in plant meiosis**
Ubiquitination	2	37	>1,300	Synapsis, DSB repair, chromosomal segregation, microtubule organization, DNA methylation, formation of telomere bouquet
Rubylation	1	1	1	CO distribution, synapsis, DNA methylation, transcription
Sumoylation	1	1	3	DSB repair, chromosome segregation, transcription

Methyl methanesulfonate sensitivity gene21 (MMS21)/high ploidy 2 (HPY2) is a conserved SUMO E3 ligase, one of three identified in *Arabidopsis* (Roy and Sadanandom, 2021), which interacts with structural maintenance of chromosome (SMC) 5 as part of the SMC5/6 complex (Liu et al., 2014; Yuan et al., 2014). Plants expressing mutant *mms21*-1 exhibited severe semi-sterility, with only 22% of the WT seed set (Liu et al., 2014). This phenotype was linked to defects in both male and female gametogenesis (Liu et al., 2014). In *mms21-1* mutant anthers, fragmented chromosomes and chromosome bridges between bivalents were observed in anaphase I, while in anaphase II sister chromatids did not segregate normally (Liu et al., 2014). Further, transcript abundance was significantly altered in *mms21-1* mutant flower buds, with *SPO11-1*, *RAD51*, *RBR*, condensin, cohesin, *SWI1*, SMC5/6 complex, and SMC-like genes showing up-regulation in the mutant; while expression of both *ASY1* and ZYP1a was reduced (Liu et al., 2014). Yuan et al. (2014) demonstrated hypersensitivity of *mms21* mutants to DNA damage, and the apparent involvement of this SUMO ligase in DSB repair by homologous recombination, indicating that unrepaired DSBs may explain the aberrant chromosome observed in *mms21-1* mutants (Liu et al., 2014). The N terminus of AtMMS21 interacts directly with the dimerization domain containing C terminus of DPa—which forms transcription factor complexes with E2F—resulting in its SUMOylation (Liu et al., 2016). The interaction of AtMMS21 with DPa abrogates its interaction with E2F and disrupts the nuclear translocation of E2Fa/DPa (Liu et al., 2016). E2Fa is one of three canonical E2Fs in *Arabidopsis* which play an essential but redundant role in both male and female gametogenesis, particularly pollen mitosis and megaspore mother cell to archesporial cell transition respectively (Yao et al., 2018).

## Methods for Identifying E3 Ubiquitin Ligase Substrates

The difficulty of identifying E3 ligase-substrate interactions is thoroughly outlined by Iconomou and Saunders (2016). In brief, the highly dynamic nature of ubiquitination and rapid degradation of many substrates presents a very brief window in which to capture the interaction between ligase and substrate (Iconomou and Saunders, 2016). Additionally, the extraordinary diversity of substrate fates and the complicated redundancy this diversity entails confounds the inference of cause and effect in mutation and knockdown studies (Iconomou and Saunders, 2016). Although putative meiotic substrates of the APC/C (PANS1 and SWI1) and SCF^*CFK*1^ (DRM2) have been recently identified in *Arabidopsis* (Cromer et al., 2019; Chen et al., 2020; Yang et al., 2020), it’s not yet clear that these interactions are conserved in other plant species nor whether they represent only a fraction of the total proteins targeted by these E3 ligase complexes. Putative substrates of SCF^*ZYGO*1^, SCF^*MOF*^—even HEI10—remain to be identified and/or substantiated in plants (He et al., 2016; Zhang et al., 2017; Ziolkowski et al., 2017; Li Y. et al., 2018; Chang et al., 2019; Zhang J. et al., 2019). Generally, meiotic E3 ligase-substrate interactions are poorly characterized, particularly in plants. For comparison, the well characterized human F-box proteins β-TrCP1 and 2, which are involved in regulation of mitotic progression as part of an SCF complex, have upwards of 50 characterized substrates (Mavrommati et al., 2018; Rayner et al., 2019).

Interaction of PANS1 with the APC/C^*CDC*20^ and of CFK1 with DRM2 was demonstrated using bimolecular fluorescence (BiFC) and yeast two-hybrid (Y2H) assays (Cromer et al., 2013; Chen et al., 2020) with later corroboration of the APC/C^*CDC*20^- PANS1 interaction *via* PANS1 pulldown and mass spectrometry and disruption of the PANS1 D and KEN-box domains (Cromer et al., 2019). Evidence for APC/C mediated degradation of SWI1 is remarkably thorough, shown *in vitro* by persistence of SWI1 lacking all five D-box motifs (2 canonical RxxLxxxxN motifs; three motifs with the minimally required RxxL) far beyond prophase I meiocytes and into tetrads (Yang et al., 2019). This was further supported by persistence of purified C-terminal SWI1 in a cell free system with: inhibition of the proteasome; abolition of SWI1 phosphorylation sites; and CDK inhibition (Yang et al., 2019). For each of these supported interactions researchers worked backwards from the characterization of a target protein to the identification of an E3 ligase responsible for mediating its degradation. Given the apparently enhanced role of ubiquitination in regulating meiosis (Tang et al., 2010; Yang et al., 2011; Yuan et al., 2018; Barakate et al., 2021), working in the opposite direction, from E3 ligase to substrates, may present an opportunity to uncover novel meiotic proteins and mechanics by identifying the substrates of ligases whose involvement in meiosis is known or implicated. The inherent challenges of this approach may be partly overcome with a growing retinue of mass spectrometry based proteomic methods.

A common method for identifying candidate E3 ligase substrates is to compare the total complement of ubiquitinated proteins in wild type cells with cells overexpressing the ligase or in which ligase function is perturbed. One method of collecting this profile is overexpression of hexa-histidine tagged ubiquitin (His_6_-Ub) followed by Ni-NTA pulldown (Beers and Callis, 1993; Saracco et al., 2009). This approach was first demonstrated by recovery of polyubiquitinated proteins with Ni^2+^ ion affinity chromatography after addition of purified His_6_-Ub to a wheat germ lysate. It was later demonstrated that His_6_-Ub could replace wild-type ubiquitin expression in yeast and that His_6_-Ub modified to prevent polyubiquitin chain formation could be expressed in *Arabidopsis* to improve recovery of ubiquitinated proteins (Ling et al., 2000). Song et al. (2011) adapted this approach to the identification of substrates by parallel overexpression of an E3 ligase (BRCA1) and His_6_-Ub followed by mass spectrometry to identify proteins which incorporated more His_6_-Ub upon E3 overexpression. A similar approach was used to capture the first SUMOylome in *Arabidopsis*, consisting of 357 putative targets (Miller et al., 2010). However, modification and/or overexpression of ubiquitin might result in atypical substrate ubiquitination (Hjerpe et al., 2009). An alternative method relies on immunoprecipitation of the characteristic di-glycine (di-gly) residue which is left attached to ubiquitinated substrate lysine residues following trypsin digestion (Xu et al., 2010). This allows enrichment of ubiquitinated proteins without potential interference from modification of ubiquitin (Xu et al., 2010). However, proteins modified by ubiquitin-like proteins SUMO and RUB/NEDD8 also leave the characteristic di-gly residue following trypsin digestion (Xu et al., 2010). Akimov et al. (2018) generated an antibody which recognizes the 13 C-terminal amino acids of ubiquitin which are retained on ubiquitinated peptides following LysC digestion. This enables ubiquitin-specific peptide enrichment in a similar manner to di-gly enrichment (Akimov et al., 2018). As Iconomou and Saunders (2016) highlight, the amount of input lysate required for di-gly enrichment may be prohibitive in some systems. Yet, with improvements in mass spectrometry van der Wal et al. (2018) reported significant overlap in identified peptides whether using 4 or 40 mg of input to each trypsin digest. Di-gly affinity purification has recently been used to profile ubiquitination during maize seed de-etiolation, using 5 mg of leaf derived protein per sample (Wang et al., 2019). Zhu et al. (2020) used di-gly affinity purification to profile the meiotic ubiquitinome in young rice panicles, identifying 916 unique proteins with approximately 100 mg of protein as input. Rose et al. (2016) reported coupling of di-gly enrichment with isobaric tagging and fractionation using a high-pH reversed-phase spin cartridge to enable multiplexed quantification of ubiquitinated peptides with only 1 mg of lysate from each of ten cell culture samples or from 7 mg of tissue culture. Isobaric tagging—labeling of peptides with unique chemical groups of identical mass, allowing peptide samples to be combined in a single MS run (Ross et al., 2004)—allows a reduction in the amount of peptide input required for capture by immunoprecipitation and reduces missing values in MS data output (Rose et al., 2016). However, isobaric tagging of peptide samples inhibits di-gly pulldown because chemical tagging of the di-gly remnant prevents interaction of the di-gly antibody and remnant motif (Rose et al., 2016). Isobaric tagging of di-gly captured peptides following elution from the antibody circumvented this problem (Rose et al., 2016). Udeshi et al. (2020) developed a similar di-gly antibody capture based method which they termed UbiFast. The main distinction between these two methods is the stage at which the isobaric tandem mass tag (TMT) is used to label the peptides (Rose et al., 2016; Udeshi et al., 2020). The UbiFast approach hypothesized that by labeling the di-gly captured peptides while still bound to the antibody instead of after elution would lead to improved yield (Udeshi et al., 2020). Indeed, in a head-to-head comparison on-antibody isobaric tagging led to an increase in the relative yield of di-gly peptides of 35.5% (Udeshi et al., 2020). This enabled quantification of more than 11000 peptides from only 0.5 mg of tumor tissue per sample (Udeshi et al., 2020). Recently, Hansen et al. (2021) coupled di-gly proteomics with tandem mass spectrometry operating in the data-independent acquisition (DIA) mode. DIA mode tandem mass spectrometry results in unbiased fragmentation of all ionized compounds in a sample based on relatively wide mass to charge windows (*m/z*), recording ion mass spectra irrespective of peptide precursor ion detection (Ludwig et al., 2018). Using this approach 89,650 di-gly sites were detected representing the deepest di-gly proteome to date (Hansen et al., 2021).

**FIGURE 6:**
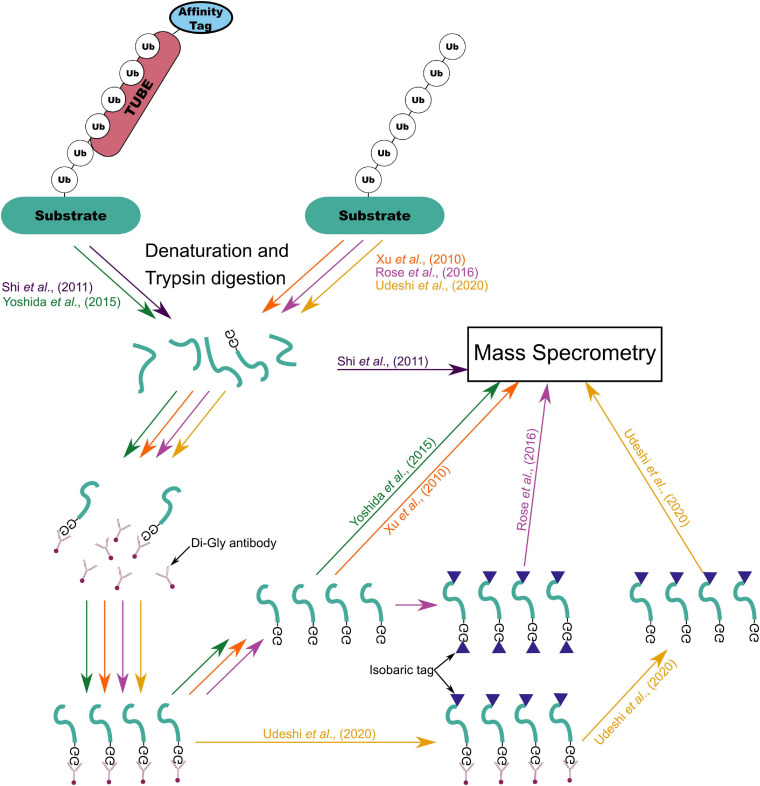
Summary of mass-spectrometry based ubiquitylome profiling workflows from Xu et al. (2010), Shi et al. (2011), Yoshida et al. (2015), Rose et al. (2016), and Udeshi et al. (2020).

Yet another approach to global ubiquitome profiling is the use of tagged tandem ubiquitin binding entities (TUBEs) to capture polyubiquitinated proteins from lysates (Hjerpe et al., 2009). TUBEs are constructed from affinity tagged tandem repeats of ubiquitin associated (UBA) domains from ubiquilin 1 and human HR23A (Hjerpe et al., 2009). Four tandem UBA domains are included based on the hypothesis that at a ubiquitin chain length of at least four is required for proteasomal degradation (Thrower et al., 2000; Hjerpe et al., 2009). Each UBA domain retains independent capacity to bind ubiquitin but in tandem dissociation of ubiquitinated proteins is reduced 1,000-fold compared to equivalent single UBA domains (Hjerpe et al., 2009). Further, TUBEs do not bind NEDD8/RUB or SUMOylated protein and the association of polyubiquitinated proteins to TUBEs protects them from DUBs and proteasomal degradations at an equivalent level to specific inhibitors (Hjerpe et al., 2009). TUBE capture was first adapted to the identification of ubiquitinated peptides using mass-spectrometry by Shi et al. (2011). Yoshida et al. (2015) generated a trypsin resistant (TR)-TUBE by substituting three arginine residues for alanine residues in tandem repeated ubiquilin 1 UBA domains. Combining expression of TR-TUBEs with subsequent di-gly enrichment significantly reduced the proportion of identified peptides which did not contain the di-gly residue when compared to di-gly alone (Yoshida et al., 2015). All of these methods allow for the enrichment of ubiquitinated proteins from whole protein extracts (Figure 6). However, for the identification of specific E3 ligase substrates where there is redundancy in ligase-substrate interactions and/or low substrate abundance they may not be suitable. An alternative approach which circumvents this issue is to introduce E3 ligase specific traps or labeling.

**FIGURE 7:**
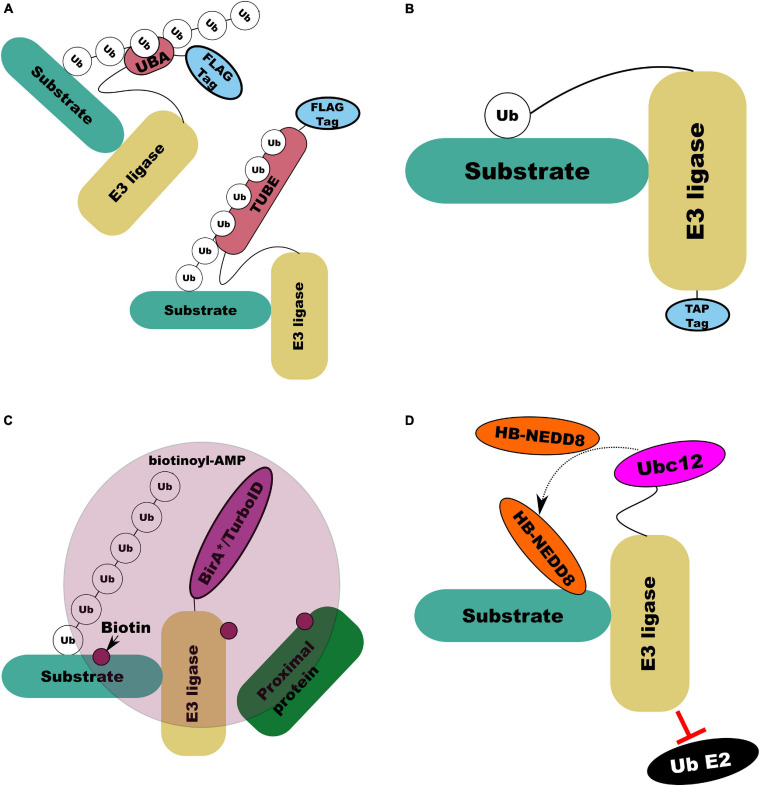
Illustrations of E3 ligase substrate capture and labeling methods. **(A)** UBA (top left) and tandem UBA (TUBE; bottom right) capture by increasing the affinity of the E3 ligase modified with the UBA or TUBE domain for its ubiquitinated substrates. **(B)** UBAIT capture forming a covalent E3-substrate complex through C-terminal modification of the E3 itself with ubiquitin *via* a flexible linker. **(C)** BioID/TurboID based proximity labeling in which modified BirA enzyme conjugated to an E3 ligase of interest results in biotin labeling of interacting and associated proteins by creating a pool of reactive biotinoyl-AMP. **(D)** NEDDylator capture, in which an E3 ligase with E2 ubiquitin interaction domain disrupted to prevent substrate ubiquitination is fused to NEDD8 conjugating E2 (Ubc12). With co-expression of his-biotin (HB) tagged NEDD8 this enables HB-NEDD8 tagging of E3 ligase substrates.

Tan et al. (2013) devised the parallel adapter capture (PAC) method which combined parallel affinity purification of HA-tagged E3 ligases expressed in cells which were untreated or were treated with either a proteasomal inhibitor or cullin ring ligase (CRL) inhibitor. This approach then combined mass-spectrometry with the Comparative Proteomics Analysis Software Suite (CompASS) to identify high confidence interacting proteins by comparison of average peptide spectral matches, a proxy for abundance, across treatments (Tan et al., 2013). By design, this approach does not specifically capture E3 ligase substrates but all proteins with which an E3 ligase might interact (Tan et al., 2013). Additionally, substrates not targeted for degradation by the ligase are unlikely to be influenced by the inhibitor treatment. Further, this approach is still confounded by the weak and transient nature of ligase-substrate interactions. Several solutions to this have been developed. Mark et al. (2016) developed ligase-traps which combined a common affinity (FLAG) tag with a UBA domain to increase the affinity of the modified ligase for its substrates, improving recovery of interacting proteins (Figure 7A). To improve recovery of ligase substrates rather than all interactors Mark et al. (2016) combined expression of their UBA-FLAG tagged E3 ligase with His_6_-Ub, allowing initial immunoprecipitation under native conditions followed by Ni^2+^ ion chromatography under denaturing conditions. An important consideration for this technique is the preference of UBA domains for ubiquitin chain linkage types; Rad23 has a fourfold preference for lys48 chains over lys68 (Mark et al., 2016). An alternative to the dual expression of modified ligase and ubiquitin to specifically recover substrates is ubiquitin-activated interaction traps (UBAITs) in which an affinity tagged E3 ligase is C-terminally tagged with ubiquitin through a flexible linker (Figure 7B; O’Connor et al., 2015). The attached ubiquitin can interact with E1 and E2 enzymes, the attached E3 facilitating recognition of its substrates and covalent attachment of the C-terminal ubiquitin to the target (O’Connor et al., 2015). The length of this linker can affect the efficiency of capture with longer linkers (up to 5xGGSG) proving more efficient at capture (O’Connor et al., 2015).

An approach which combines ligase-substate trapping with TUBE and di-gly was recently developed by Watanabe et al. (2020). This approach replaces the UBA-FLAG tagged E3 ligase proposed by Mark et al. (2016) with a TUBE-FLAG tag, further increasing the affinity of the ligase for its substrate and protecting the substrate from degradation (Figure 7A) (Watanabe et al., 2020). The addition of di-gly enrichment following anti-FLAG immunoprecipitation and trypsin digest of lysates lead to a dramatic increase in efficiency of putative substrate capture (Watanabe et al., 2020). Watanabe et al. (2020) also highlighted that attachment of the TUBE bait tag to the N or C terminal of the ligase affected the efficiency of capture in a ligase dependant manner. In *Arabidopsis*, Durand et al. (2016) and Lee et al. (2018) expressed affinity tagged RING and F-box proteins respectively which retained their ability to interact with substrates but lacked the ability to ligate ubiquitin. These substrate trapping approaches all rely on the ability to express modified proteins in a system of interest which may be prohibitive. In addition, as with His_6_-Ub, modification or overexpression of substrates might generate non-native interactions. However, by specifically targeting E3 ligase substrates they offer a way to dramatically limit the depth of proteomic profiling required to identify putative substrates. Further, because they do not rely on assessing the stability of substrates, they can be more effective in identifying redundant and non-degradative interactions.

Another general approach, developed by Roux et al. (2012), which is not selective for specific substrates, is proximity-dependant biotin labeling (BioID), in which a protein of interest is fused to a mutant form of *E. coli* biotin conjugating enzyme BirA (BirA^∗^) which is defective in self-association and DNA binding (Figure 7C). BirA^∗^ can activate biotin, generating biotinoyl-AMP, but its affinity for the activated substrate is two orders of magnitude lower than wild type BirA, allowing biotinoyl-AMP to interact with nearby amines, covalently modifying proteins near to the modified peptide with biotin which can then be purified with streptavidin (Roux et al., 2012). Coyaud et al. (2015) deployed BioID to characterize over 50 putative interactors for β-TrCP. However, the lengthy (16–24 h) incubation at high temperature (37°C) required for efficient BioID labeling is not optimal for *in vivo* proximity labeling in plants (Zhang Y. et al., 2019). Branon et al. (2018) engineered BirA to produce promiscuous mutants capable of proximity labeling with biotin in only 10 min which they called TurboID and miniTurbo. Zhang Y. et al. (2019) deployed TurboID to determine the interactions of an immune receptor in *Nicotiana benthamiana*, demonstrating that TurboID at room temperature was significantly more efficient than BioID at 37°C. Recently, Wu et al. (2020) replicated this approach to identify specific E3 ligase interactions through expression of TurboID-tagged E3 ligases SNIPER1 and SNIPER2 in *N. benthamiana*. A similar proximity labeling approach has been developed by Zhuang et al. (2013) in which histidine-biotin (HB) tagged (Tagwerker et al., 2006) NEDD8/RUB E2 equivalent enzyme (Ubc12) (Figure 7D) is linked to a RING E3 ligase of interest with its RING domain removed to prevent its interaction with ubiquitin E2 conjugating enzymes. Expression of this construct *in vivo* or its addition to cell lysate leads to stable, covalent labeling of E3 ligase targets with RUB/NEDD8 which can be purified by both Ni^2+^ ion and streptavidin chromatography (Zhuang et al., 2013).

The difficulty of investigating E3-substrate interactions in plant meiosis is further compounded by the challenge of capturing enough meiotic cells at the right time. Plant meiotic cells are scarce and are embedded in complex tissues comprised largely of vegetative cells. In barley, meiocytes account for only 10% of cells in the developing anther (Lewandowska et al., 2019). A common strategy to overcome this is to collect meiotic tissues in bulk. This approach—while time consuming—has produced many valuable large scale meiotic transcriptomic and proteomic data sets. Several *Arabidopsis* studies have used this approach despite that *Arabidopsis* meiocytes have a diameter of only about 5 microns (Yang et al., 2011; Chen and Retzel, 2013). Similar methods have been developed in maize, wheat, and brassica (Greer et al., 2012; Khoo et al., 2012; Dukowic-Schulze et al., 2014; Osman et al., 2018). In rice, collection of approximately 10,000 anthers between PMCs to microspores allowed profiling of both the proteome (Collado-Romero et al., 2014; Ye et al., 2015) and acetylated proteins (Li X. et al., 2018). Non-destructive methods of approximating meiotic stage, such as by anther length (Arrieta et al., 2020), can reduce the labor intensity of such methods. The transient nature of E3-substrate interactions and ordered progression of meiosis might require isolation of meiotic tissue at a precise stage of development. Bulk collection of meiotic tissue can introduce variation in even carefully staged samples which may obscure small or rapid changes. Further, development of meiocytes within the same anther may not be fully synchronized but it is possible to overcome this issue by introducing further staging steps such as cytological analysis (Shunmugam et al., 2018; Barakate et al., 2021). Recently, Lewandowska et al. (2019) developed a micro-proteomic workflow, allowing identification of ∼2,800 and 4,000 proteins from precisely staged single and paired barley anthers. The amount of ubiquitinated compared to non-ubiquitinated protein at any one time is low (Hristova et al., 2020). As such comparative ubiquitylomics is outside the reach of micro-proteomics at the time of writing. However, as direct substrate capture methods do not require deep profiling they could be applied to smaller samples such as fewer anthers or isolated meiocytes, increasing the practicality of highly accurate staging.

## Outlook

While identification of the E3 ligases and their substrates involved in meiosis in plants remains a substantial undertaking, there are related processes worthy of exploration. In humans, the E3/E4 ligase UBE4A has been shown to be required for optimal DSB repair through fine adjustment of both K48 and K63-linked ubiquitin chain lengths in protein complexes involved in DSB repair (Baranes-Bachar et al., 2018). In the *C. elegans* germ line, the E4 ubiquitin ligase ubiquitin fusion degradation-2 (UFD-2) ensures the timely removal of RAD-51 from DSB sites and is involved in regulating the apoptotic response in the germ line when meiotic recombination intermediates or DSBs persist in late pachytene stage (Ackermann et al., 2016). An *Arabidopsis* ortholog of UFD-2, UBE4/Mutant, SNC1-Enhancing 3 (MUSE3), is known to be involved in the tight regulation of immune receptor degradation (Huang et al., 2014; Skelly et al., 2019). It is possible that, in addition to its role in the immune response, it might also play a role in regulation of DSB repair in plants. More broadly, it is possible that, given the complex temporal and spatial organization of meiotic processes, some meiotic ubiquitination events may operate in a “dimmer switch” rather than binary on/off manner as has been observed in other processes (Skelly et al., 2019). The essential reversing component to such a system are the DUBs (Skelly et al., 2019). Meiotic transcriptomes in both barley (Barakate et al., 2021) and *Arabidopsis* (Yang et al., 2011) point to enrichment of DUBs during male meiosis. The highly homologous ubiquitin specific proteases (UBPs) UBP3 and UBP4 have been shown to regulate pollen development at various stages in *Arabidopsis* (Doelling et al., 2007). Enrichment of DUBs in Barley anthers at pachytene–diplotene stages was driven by four ovarian tumor domain (OTU) proteases (Barakate et al., 2021), unfortunately these are presently poorly characterized in plants (March and Farrona, 2018). DUBs are fewer in number and exhibit less specificity than E3 ligases (Li et al., 2020); however, similar approaches can be deployed in characterizing their substrates (Sowa et al., 2009).

## Author Contributions

JO led manuscript preparation. RW and IC contributed to manuscript revision, read, and approved the submitted version. All authors contributed to the article and approved the submitted version.

## Conflict of Interest

The authors declare that the research was conducted in the absence of any commercial or financial relationships that could be construed as a potential conflict of interest.

## Glossary

**Table:**

AGG11	ABERRANT GAMETOGENESIS 1
AM11	AMEIOTIC 1
ASKK	ARABIDOPSIS SKP1-LIKE
ASY11	ASYNAPTIC 1
ASY33	ASYNAPTIC 3
AXR11	AUXIN RESISTANT 1
ββ-TrCPP	BETA-TRANSDUCIN REPEATS-CONTAINING PROTEIN 1
BirAA	BIFUNCTIONAL LIGASE/REPRESSOR A
BRCA11	BREAST AND OVARIAN CANCER SUSCEPTIBILITY
	PROTEIN 1
CAP-D33	CONDENSIN-2 COMPLEX SUBUNIT D3
CCS522	CELL CYCLE SWITCH PROTEIN 52
CDC200	CELL DIVISION CYCLE 20
CFK11	COP9 SIGNALOSOME INTERACTING F_BOX KELCH 1
CDKK	CYCLIN DEPENDANT KINASE
COI11	CORONATINE INSENSITIVE 1
CUL11	CULLIN 1
DMC11	DISRUPTED MEIOTIC CDNA 1
DPaa	DIMERIZATION PARTNER A
DRM22	DOMAINS REARRANGED METHYLTRANSFERASE2
E2FF	E2 FACTOR
EST11	EVER SHORTER TELOMERES PROTEIN 1
HEI100	HUMAN ENHANCER OF INVASION 10
HEIP11	HEI10 INTERACTION PROTEIN
HPY22	HIGH PLOIDY 2
HR23AA	HOMOLOGUE OF RAD23 A
JMJ166	JMJC DOMAIN-CONTAINING PROTEIN 16
MER33	MEIOTIC RECOMBINATION 3
MLH33	MUTL HOMOLOG 3
MMD11	MALE MEIOCYTE DEATH 1
MMS211	METHYL METHANESULFONATE SENSITIVITY GENE 21
MOFF	MEIOTIC F-BOX
MSH44	MUTS HOMOLOG 4
MSH55	MUTS HOMOLOG 5
MUSE33	MUTANT, SNC1-ENHANCING 3
OSD11	OMISSION OF SECOND DIVISION 1
OSK11	ORYZA SATIVA SKP1-LIKE
PANS11	PATRONUS 1

**Table:**

PCH22	PACHYTENE CHECKPOINT PROTEIN 2
PDS55	PRECOCIOUS DISSOCIATION OF SISTER 5
PTDD	PARTING DANCERS
RAD511	RADIATION-SENSITIVE 51
RAD233	RADIATION-SENSITIVE 23
RNF2122	RING FINGER PROTEIN 212
RSS11	RICE SALT SENSITIVITY1
SGOO	SHUGOSHIN
SHOC11	SHORTAGE IN CHIASMATA 1
SINAA	SEVEN *IN ABSENTIA*
SMC11	STRUCTURAL MAINTENANCE OF CHROMOSOMES 1
SMC33	STRUCTURAL MAINTENANCE OF CHROMOSOMES 3
SMC55	STRUCTURAL MAINTENANCE OF CHROMOSOME 5
SMC66	STRUCTURAL MAINTENANCE OF CHROMOSOME 6
SMG77	SUPPRESSOR WITH MORPHOGENETIC EFFECTS ON
	GENITALIA 7
SNIPER 11	SNC1-INFLUENCING PLANT E3 LIGASE REVERSE 1
SNIPER 22	SNC1-INFLUENCING PLANT E3 LIGASE REVERSE 2
SUMOO	SMALL UBIQUITIN-LIKE MODIFIER
SWI11	SWITCH 1
SYCP22	SYNAPTONEMAL COMPLEX PROTEIN 2
SYP33	SYNAPTONEMAL COMPLEX PROTEIN 3
TAMM	TARDY ASYNCHRONUS MEIOSIS
TDM11	THREE DIVISION MUTANT 1
TEX111	TESTIS-EXPRESSED GENE 11
TOPIII	TOPOISOMERASE II
TRIP133	THYROID RECEPTOR-INTERACTING PROTEIN 13
UBC222	UBIQUITIN CONJUGATING ENZYME E2 22
UBE2SS	UBIQUITIN CONJUGATING ENZYME E2 S
UBE2CC	UBIQUITIN CONJUGATING ENZYME E2 C
UBE4AA	UBIQUITINATION FACTOR E4A
UBP3/44	UBIQUITIN SPECIFIC PROTEASE3/4
UFD-22	UBIQUITIN FUSION DEGRADATION-2
WAPLL	WINGS APART-LIKE
ZYGO11	ZYGOTENE1
ZYP11	MOLECULAR ZIPPER 1-LIKE PROTEIN
ZIP33	MOLECULAR ZIPPER PROTEIN 3
ZIP44	MOLECULAR ZIPPER PROTEIN 4
